# Retraction of the landmark glyphosate safety publication by Williams, Kroes and Munro (2000) should be reversed

**DOI:** 10.17179/excli2026-9644

**Published:** 2026-07-14

**Authors:** Christopher J. Borgert, Mohammad Abdollahi, Jay M. Ansell, Michael Aschner, Christopher A. Bates, Sir Colin Berry, Brad Bolon, Peter J. Boogaard, Robert A. Budinksy, Lyle D. Burgoon, Gary Burin, James S. Bus, Harvey Clewell, Samuel M. Cohen, Thomas Colnot, Paul S. Darby, Wolfgang Dekant, John DeSesso, Daniel R. Dietrich, Mirjana Djukic, Anca Oana Docea, Jose L. Domingo, David L. Eaton, Jeffery A. Engelhardt, Anne Fairbrother, Allan Felsot, Penelope A. Fenner-Crisp, Robinan Gentry, Jay I. Goodman, Gio B. Gori, Helmut Greim, J.S. Heslop-Harrison, Jan G. Hengstler, Hartmut Jaeschke, Norbert E. Kaminski, Sam Kacew, James Kim, Anthony L. Kiorpes, Curtis Klaassen, James E. Klaunig, Anne Loccisano, James S. MacDonald, Angela Mally, Hans W. J. Marquardt, M. Sue Marty, John C. Matthews, Roger O. McClellan, Robert G. Meeks, Glenn C. Millner, Michael W. Pariza, Dennis Paustenbach, Olavi Pelkonen, David Sarvadi, Kai Savolainen, Ted W. Simon, Carr J. Smith, Keith R. Solomon, Stanley M. Tarka, Jr., Aristidis M. Tsatsakis, Joyce Tsuji, Nico P. E. Vermeulen, Kendall B. Wallace, Michael Wernke, Jonathan P. Williams, Raphael J. Witorsch, Hiroshi Yamazaki

**Affiliations:** 1 Applied Pharmacology & Toxicology, Inc., Gainesville, FL, USA; 2 Mohammad Abdollahi, Department of Toxicology and Pharmacology, Tehran University of Medical Sciences, Tehran, Iran; 3 Jay M. Ansell, Principal, Bellevue Toxicology LLC, Alexandria VA, USA; 4 Michael Aschner, Department of Molecular Pharmacology, Albert Einstein College of Medicine, Bronx, NY, USA; 5 Christopher A. Bates, H.B. Fuller, Saint Paul, MN, USA; 6 Sir Colin Berry, Prof. (emeritus) of Pathology, Queen Mary, London, UK; 7 Brad Bolon, Sr. Advisor to the Editor-in-Chief of the journal Toxicologic Pathology, President and Pathologist, GEMpath, Inc., Longmont, CO, USA; 8 Peter J. Boogaard, Editor-in-Chief of Critical Reviews in Toxicology, Professor of Environmental Health & Human Biomonitoring, Division of Toxicology - AFSG, Wageningen University and Research, Wageningen, The Netherlands; 9 Robert A. Budinsky, (retired) The Dow Chemical Company, Midland, MI, USA; 10 Lyle D. Burgoon, Raptor Pharm & Tox, Ltd. Apex, NC, USA; 11 Gary Burin, (retired) Technology Sciences Group, Inc., Vienna, VA, USA; 12 James S. Bus, Exponent, Inc., Wendell, NC, USA; 13 Harvey Clewell, Science to Support Regulatory Activities, Ramboll Americas Engineering Solutions, Inc., Research Triangle Park, NC, USA; 14 Samuel M. Cohen, Department of Pathology, Microbiology and Immunology, College of Medicine, University of Nebraska Medical Center, Omaha, NE, USA; 15 Thomas Colnot, EUROTOX, CiS Toxicology, Osorno, Chile; 16 Paul S. Darby, MD PhD Services, PLLC, Tacoma, WA, USA; 17 Wolfgang Dekant, Prof. (emeritus) of Toxicology, Department of Pharmacology and Toxicology, University of Würzburg, Germany; 18 John DeSesso, Exponent, Inc., Alexandria, VA, USA; 19 Daniel R. Dietrich, Prof. (emeritus) of Toxicology, Department of Human and Environmental Toxicology, University of Konstanz, Germany, Past-President Academy of Toxicological Sciences, Editor-in-Chief of Chemico-Biological Interactions, Computational Toxicology, and the Journal of Toxicology and Regulatory Policy, Konstanz, Germany; 20 Mirjana Djukic, University of Belgrade-Faculty of Pharmacy, Department for Toxicology, Belgrade, Serbia; 21 Anca Oana Docea, Department of Toxicology, University of Medicine and Pharmacy of Craiova, Craiova, Romania; 22 Jose L. Domingo, Distinguished Prof. (emeritus) of Toxicology, Universitat Rovira i Virgili, School of Medicine, Reus, Spain; 23 David L. Eaton, President, Academy of Toxicological Sciences, Prof. (emeritus) School of Public Health, University of Washington, Seattle, WA, Adjunct Professor of Pharmacology & Toxicology, R. Ken Coit College of Pharmacy, University of Arizona, Tucson, AZ, USA; 24 Jeffery A. Engelhardt, (retired) Vice President of Pathology and Nonclinical Development, Ionis, Carlsbad, CA, USA; 25 Anne Fairbrother, (retired), Exponent, Inc., Philomath, OR, USA; 26 Allan Felsot, Department of Entomology & Environmental Toxicology, Washington State University, Richland, WA, USA; 27 Penelope A. Fenner-Crisp, (retired) Environmental Protection Agency, Charlottesville, VA, USA; 28 Robinan Gentry, Science to Support Regulatory Activities, Ramboll Americas Engineering Solutions, Inc., Monroe, LA, USA; 29 Jay I. Goodman, Prof. (emeritus), Department of Pharmacology and Toxicology, Institute for Integrative Toxicology, Michigan State University, East Lansing, MI, USA; 30 Gio B. Gori, Editor-in-Chief (emeritus) of Regulatory Toxicology and Pharmacology(2002 - 2009), The Health Policy Center, Bethesda, MD, USA; 31 Helmut Greim, Prof. (emeritus) of Toxicology, Technical University of Munich, Germany; 32 J.S. Heslop-Harrison, Genetics and Genome Biology, Institute for Environmental Futures, University of Leicester, UK; 33 Jan G. Hengstler, Department of Toxicology, Leibniz Research Centre for Working Environment and Human Factors, Dortmund, Germany; 34 Hartmut Jaeschke, Editor-in-Chief of Livers, Associate Editor of Toxicological Sciences, Distinguished Prof. Department of Pharmacology, Toxicology & Therapeutics, University of Kansas, Kansas City, KS, USA; 35 Norbert E. Kaminski, Department of Pharmacology & Toxicology, Michigan State University East Lansing, MI, USA; 36 Sam Kacew, Editor-in-Chief of Journal of Toxicology and Environmental Health Parts A & B, McLaughlin Centre for Population Health Risk Assessment, Faculty of Medicine, University of Ottawa, ON, Canada; 37 James Kim, Sr. Vice President, Science & Regulatory Affairs, American Cleaning Institute®, Washington, DC, USA; 38 Anthony L. Kiorpes, Principal, River Bluff Associates, LLC, Bloomington, MN, USA; 39 Curtis Klaassen, (retired) Department of Pharmacology, Toxicology and Therapeutics, College of Medicine, University of Kansas, Kansas City, KS, USA; 40 James E. Klaunig, Prof. (emeritus) of Environmental Health and Toxicology, Indiana University, Bloomington, IN, USA; 41 Anne Loccisano, Exponent, Inc., Alexandria, VA, USA; 42 James S. MacDonald, Founding Partner, Synergy Partners R&D Solutions, Chester, NJ, USA; 43 Angela Mally, Department of Toxicology, University of Würzburg, Germany; 44 Hans W. J. Marquardt, Prof. (emeritus) of Toxicology, Department of Experimental and Clinical Pharmacology and Toxicology, University of Hamburg Medical School, Germany; 45 M. Sue Marty, The Dow Chemical Company, Midland, MI, USA; 46 John C. Matthews, Prof (emeritus) University of Mississippi School of Pharmacy, University, MS, USA; 47 Roger O. McClellan, Editor-in-Chief (emeritus) of Critical Reviews in Toxicology (1987 - 2025); Independent Advisor, Toxicology and Human Health Risk Analysis, Albuquerque, NM, USA; 48 Robert G. Meeks, Shin-Etsu Silicones of North America, Sarasota, Florida, USA; 49 Glenn C. Millner, Founder and Principal Toxicologist, Center for Toxicology and Environmental Health (CTEH), LLC, Little Rock, AR, USA; 50 Michael W. Pariza, Prof. (emeritus), Food Science, Director (emeritus), Food Research Institute University of Wisconsin-Madison, WI, USA; 51 Dennis Paustenbach, Toxicology and Risk Exposure, TRC, Jackson, WY, USA; 52 Olavi Pelkonen, Prof. (emeritus) of Pharmacology, Department of Pharmacology and Toxicology, University of Oulu, Finland; 53 David Sarvadi, MS, JD, Bonita Springs, FL, USA; 54 Kai Savolainen, Editor (emeritus) of Human and Experimental Toxicology (2002-2024), Finnish Institute of Occupational Health, Helsinki, Finland; 55 Ted W. Simon, Ted Simon LLC, College of Public Health, University of Georgia, Winston, GA, USA; 56 Carr J. Smith, Society for Brain Mapping and Therapeutics, Mobile Alabama, USA; 57 Keith R. Solomon, Prof. (emeritus), School of Environmental Sciences, University of Guelph, Guelph, ON, Canada; 58 Stanley M. Tarka, Jr., Adjunct Associate Professor, Neuroscience and Experimental Therapeutics, Pennsylvania State University College of Medicine, Hershey, PA, USA; 59 Aristidis M. Tsatsakis, Center of Toxicology and Science Applications, Medical School, University of Crete, Heraklion, Greece; Universidad Ecotec, Health Sciences Dept., Km. 13.5 Samborondón, EC092302, Ecuador; Sechenov IM First State Medical University, Science Technology Park, Moscow 119991, Russia; 60 Joyce Tsuji, Exponent, Inc., Bellevue, WA, USA; 61 Nico P. E. Vermeulen, Prof. (emeritus) of Molecular Toxicology, Department of Chemistry & Pharmaceutical Sciences, VU University Amsterdam, The Netherlands; 62 Kendall B. Wallace, Prof. (emeritus) of Biochemistry & Molecular Biology, University of Minnesota Medical School, Duluth, MN, USA; 63 Michael Wernke, Health Sciences Sr. Toxicologist and Pharmacologist, Sea Limited, Columbus, OH, USA; 64 Jonathan P. Williams, Assoc. Prof., Department of Statistics, North Carolina State University, Raleigh, NC, USA; 65 Raphael J. Witorsch, Prof. (emeritus) of Physiology and Biophysics, School of Medicine, Virginia Commonwealth University, Richmond, Virginia, USA; 66 Hiroshi Yamazaki, Showa Pharmaceutical University, Machida, Tokyo, Japan

**Keywords:** scientific integrity, retraction, COPE, editorial censorship, scientific publishing

## Abstract

The decision by the co-Editor-in-Chief of Regulatory Toxicology and Pharmacology, Prof. Martin van den Berg, to retract the 2000 review article by Williams, Kroes, and Munro has elicited widespread criticism within the scientific community. Issued in late 2025, the retraction decision cites procedural concerns including potential ghostwriting, undisclosed conflicts of interest, and omission of certain unpublished studies, invoking Committee on Publication Ethics guidelines despite lacking evidence of fraud or scientific flaws. This editorial argues that the retraction decision involves editorial overreach and misapplication of the guidelines. The alleged omissions stemmed from proprietary data access limitations that were disclosed in the original paper. Subsequent reviews by several independent expert panels and regulatory authorities with access to all glyphosate data, including the studies cited by the retracting editor, reached similar conclusions. Claims of ghostwriting were previously investigated and found lacking, including a declaration by EFSA as to the clarity of the conflict disclosures. The retraction's timing, reliance on litigation documents, and apparent biases that were not disclosed in the retraction notice raise questions of ideological interference. Absent substantive rebuttals based on scientific merit rather than speculative claims of inappropriate authorship and data access, this retraction decision sets a dangerous precedent for retroactive censorship, potentially chilling beneficial industry-academic collaborations and eroding trust in the integrity of scientific publishing. With the strongest conviction, we assert that retracting a paper without scientific flaws isn't protection-it is censorship. We therefore call for the immediate reversal of this flawed and unjustified retraction to preserve trust in peer-reviewed literature.

## ⁯⁯

- The retraction of Williams et al. (2000[29]) lacks evidence of scientific flaws or fraud, relying instead on procedural concerns and subjective 'loss of confidence' under COPE guidelines.
- Omitted proprietary studies were inaccessible due to ownership by competitors, with subsequent reviews confirming no impact on the paper's conclusions.
- Independent assessments by expert panels and regulatory agencies affirm the findings of the retracted article, highlighting the paper's scientific validity.
- Claims of ghostwriting were previously evaluated and found to lack merit.
- The retraction's timing and reliance on litigation documents suggest undisclosed ideological biases, potentially from critics with anti-industry ties, violating transparency standards.
- Reversing the retraction is essential to avoid a chilling effect on industry-academic collaborations and to preserve confidence in the integrity of scientific publishing.
- This work was first submitted on February 27, 2026, as a Commentary to *Regulatory Toxicology and Pharmacology* as a courtesy to retracting editor Dr. Martin van den Berg, the journal, and Elsevier. It was withdrawn because co-Editor-in-Chief Dr. van den Berg would only accept a truncated version that neither transparently listed all authors nor permitted a full exposition of his retraction decision.
- This work was then invited as a Guest Editorial in *Archives of Toxicology* (AoT), receiving full acceptance in March 2026. Following acceptance, publication in AoT was repeatedly and unjustifiably delayed due to inappropriate interference by the publisher of that journal (Springer Nature) over the objections of the Editor-in-Chief and Deputy Editor-in-Chief.

In a move that has sparked significant dissatisfaction within the scientific community, the journal *Regulatory Toxicology and Pharmacology *(*RTP*), published by Elsevier, Inc., recently retracted a 25-year-old review article by Gary M. Williams, Robert Kroes and Ian C. Munro, titled “Safety Evaluation and Risk Assessment of the Herbicide Roundup and its Active Ingredient, Glyphosate, for Humans” (Williams et al., 2000[29]). Published in 2000, this comprehensive review paper concluded that glyphosate, the active ingredient in Monsanto's Roundup™ herbicide, posed no significant health risks to humans based on data available at the time. The retraction notice (cited as: Williams et al., 2025[30]), was issued in late 2025 by handling co-Editor-in-Chief Prof. Martin van den Berg, who claimed ethical concerns over authorship, potential ghostwriting by Monsanto employees, undisclosed conflicts of interest, and the exclusion of certain unpublished studies. We join a growing chorus of scientists and journal editors who contend that this decision was unfounded and amounts to editorial over-reach. We strongly encourage Dr. van den Berg and Elsevier to promptly reverse this retraction.

The retraction notice invokes guidelines from the Committee on Publication Ethics (COPE), which allow editors to withdraw articles if they lose confidence in their integrity, even without definitive proof of fraud (Committee on Publication Ethics [COPE], 2011[1]). Dr. van den Berg emphasized that the action does not take a stance on glyphosate's carcinogenicity but instead stems from unaddressed concerns, including the sole surviving author's failure to respond to van den Berg's allegations. Critically, the retraction notice by van den Berg (cited as: Williams et al., 2025[30]) highlights no substantive scientific flaws in the paper's methodology or conclusions-only procedural issues, such as alleged misrepresentation of author contributions and reliance on Monsanto-sponsored, unpublished studies while omitting others-and admits no proven fraud, relying instead on subjective 'loss of confidence.' The retracting editor's rationale overlooks essential facts that undermine its validity, including the absence of any new evidence beyond Monsanto emails from 2015, which were unsealed in 2017 and already addressed in prior reviews without finding misconduct (Cornwall, 2017[4]; Malkan, 2025[17]; Mozart, 2025[21]). Prior to its retraction, the Williams et al. 2000[29], paper had been cited over 1,200 times, with its findings echoed in independent reviews by bodies like the Food and Agriculture Organization and World Health Organization (FAO/WHO). As one of the most widely-cited articles ever published in *RTP*, the lack of substantive, comprehensive, refereed scientific rebuttals to Williams et al. published in any venue is remarkable.

A central point of contention in the retraction notice is the retracting editor's criticism of the authors for not incorporating specific unpublished long-term toxicity and carcinogenicity studies in existence in 1999. The notice lists studies from companies such as Inveresk Research International and the Institute of Environmental Toxicology, suggesting that their exclusion casts doubt on the paper's objectivity. However, those studies were proprietary and owned by entities other than Monsanto, which could only provide the authors access to its own data. Monsanto could not legally or practically grant access to competitors' confidential research, making the omission a matter of straightforward access limitations rather than suspicious bias. This exclusion was disclosed in the original article. Thus, far from being a red flag, the authors' inability to include proprietary studies from other institutions reflects standard industry and journal practices at the time of publication.

Moreover, those proprietary studies from other companies were subsequently reviewed and analyzed by other authors and by several expert panels under the auspices of international agencies and regulatory authorities (ECHA, 2017[6][5], 2022[8][7]; EFSA, 2023[10]; FAO/WHO, 2004[12], 2016[13]; FSCJ, 2016[14]; Greim et al., 2015[15]; U.S. EPA, 2016[24][26][25]; Williams et al., 2016[28]). Those reviewers had access to all glyphosate data, including the studies cited by the retracting editor, and all reached similar conclusions to the Williams et al. (2000[29]) review that van den Berg retracted. For example, the 2015 review by Helmut Greim and colleagues in *Critical Reviews in Toxicology *(*CRT*) (Greim et al., 2015[15]) evaluated tumor incidence data from 14 chronic/carcinogenicity rodent studies, including the ones *RTP*'s retracting editor flagged, concluding there was "no evidence of a carcinogenic effect related to glyphosate treatment" and no plausible mechanism for such an effect. This later work directly supports the original findings of Williams et al. (2000[29]), demonstrating that including the omitted studies would not have altered the conclusions of the 2000 paper. The conclusions of Williams et al. (2000[29]) are further supported by the evaluation of the carcinogenic potential of glyphosate by four independent expert panels, published in 2016 in *CRT *(Williams et al., 2016[28]). By the end of 2024, this 2016 publication had become the most frequently downloaded paper published in *CRT *(32,466 times). The retracting editor of *RTP*, Dr. van den Berg, ignored this post-publication validation of Williams et al. (2000[29]), contrary to the corrective intent of retraction as explained in the COPE guidance (COPE, 2025[2]).

Although critics claim the Williams et al. (2000[29]) paper had a disproportionately large influence on regulatory decisions and ignored non-industry research (Marston, 2025[18]), such claims appear to misapprehend the rigorous review and assessment process used by many regulatory agencies. The European Food Safety Authority (EFSA) determined that, in this case, ghostwriting of this review would not have altered their risk assessment and that the declaration of interest within Williams et al. (2000[29]) was clear to EFSA reviewers (European Food Safety Authority [EFSA], 2017[9]). Modern assessments by agencies such as the U.S. Environmental Protection Agency (EPA), Health Canada, EFSA, and FAO/WHO Joint Meeting on Pesticide Residues (JMPR) do not rely on secondary reviews of studies, nor do they restrict their evaluations to the studies reviewed by Williams et al. in 2000[29]; instead, agencies draw on decades of subsequent data to conduct independent evaluations of chemical safety. In this instance, these independent assessments of glyphosate's safety by multiple expert panels and agencies around the globe affirm the conclusions of the retracted Williams et al. (2000[29]) article, and the retraction is irrelevant to their conclusions (Moretto and Boobis, 2026[20]).

Rather than issues of scientific merit and reproducibility, the *RTP *retraction relies heavily on litigation documents from U.S. court cases, which surfaced years ago but were only acted upon now, eight years after initial ghostwriting claims were made. This delay raises questions about timing, especially as the retraction decision appears to have been spearheaded by authors Alexander Kaurov and Naomi Oreskes (Malkan, 2025[17]; Mozart, 2025[21]). Their 2025 paper claiming that the Williams et al. 2000[29] paper was ghostwritten was funded by the Rockefeller Family Fund (Kaurov and Oreskes, 2025[16]), an organization linked to anti-industry litigation campaigns (Zaruk, 2025[32]). Neither Kaurov nor Oreskes has expertise in toxicology or cancer biology. Reports have since highlighted Oreskes' connections to the litigation industry (Zaruk, 2023[31]), including allegations of data manipulation to advance predetermined narratives against companies like Monsanto (Modern Ag Alliance, 2025[19]; Zaruk, 2025[32]). The *RTP *retraction did not disclose potential biases that may have influenced it, including those of Kaurov and Oreskes and their communications with the editor. Such omissions seem to violate the transparency standards that COPE itself promotes, turning the guidelines into a tool for selective enforcement rather than for equitable oversight.

Notably, the *RTP *retraction focuses only on cancer-related issues and studies, whereas the retracted article addressed the broader toxicological database of glyphosate and Roundup™. The focus on cancer is consistent with claims made in lawsuits against glyphosate-containing products, but not with the broad toxicological context of the Williams et al. 2000[29] paper nor with the lack of rebuttal of its scientific merit and integrity. The retraction was released on November 29, 2025, just days before the U.S. Solicitor General filed a brief supporting the appeal by Bayer (which acquired Monsanto in 2018) in Roundup™ litigation, and amid the EPA's ongoing registration review process for glyphosate, presenting an appearance of potential influence from narratives generated by ongoing regulatory and legal battles rather than principled reevaluation based on new scientific evidence (Marston, 2025[18]; Sullivan Papain, 2026[23]).

Compounding these issues is the *RTP *retracting editor's apparent lack of due diligence, which is a requirement of the COPE code of conduct for journal editors, Chapter 11 topics 11.2, 11.4, and 11.5 COPE, 2011[1]). Dr. van den Berg failed to disclose that the New York Medical College previously investigated allegations of ghostwriting for the Williams et al. (2000[29]) paper and found no support for those claims (Cornwall, 2017[4]). Dr. van den Berg apparently did not attempt to contact the scientists at Bayer who provided data access and assistance to Williams and coauthors and who are mentioned by name in the original publication, as is coauthor Munro's associate, Douglas Bryant. These individuals could have provided firsthand clarification on author roles and contributions, potentially resolving allegations of ghostwriting. Nor does it appear that Dr. van den Berg sought input from the estates of the deceased coauthors, relying instead on a single non-response by the surviving retired coauthor to justify proceeding with the retraction (Mozart, 2025[21]; Williams et al., 2025[30]). Importantly, the co-editors of *RTP *did not sign the retraction, and input from the current editorial board of *RTP *and from editorial board members who oversaw the acceptance of Williams et al. (2000[29]) for publication seems to be lacking. Co-authors of this Guest Editorial include current members of the *RTP *editorial board. The lack of vocal support by other *RTP *editorial personnel suggests that the retraction by the Co-Editor in Chief Dr. van den Berg does not reflect a majority view within the editorial board of *RTP*.

As authors, editors, peer-reviewers, and members of the editorial boards of prominent journals in various scientific fields, including the field of toxicology and the journal *RTP*, we believe that the retraction of the Williams et al. (2000[29]) review is both unjustified and indefensible. The decision was not grounded in any scientific error or fraud in the retracted article but appears to reflect a subjective ideological perspective that failed to consider and account for all the facts. This solo editorial decision sets a dangerous precedent in which historical, well-received papers are withdrawn on alleged procedural technicalities long after the standards by which they were originally accepted may have changed. Indeed, the COPE guidelines were first published in 1999 and have undergone significant revisions in 2004, 2007, 2011, and 2017, (COPE, accessed 2//20/2026[3]). Moreover, long-delayed retractions like this (25 years after the original publication) are likely to find the authors retired or no longer alive and thus in no position to defend themselves or the data they had published. Instead, the proper answer to any editorial qualms about this paper would have been to seek substantive, refereed scientific rebuttals rather than to issue a retraction with no objective evidence to justify such an action (Siddique et al., 2024[22]).

In our view, this retraction underscores even more important, broader concerns about ideological interference in scientific publishing. While COPE guidelines claim an intent to protect journal integrity, their application in the retraction of Williams et al. (2000[29]) appears to be internally inconsistent and arbitrarily selective, retroactively discrediting valid research prejudicially without offering new evidence of scientific misconduct or weakness. The decision risks a chilling effect on the traditionally vibrant industry-academic partnerships that provide many advantages and benefits from robust data access in toxicology (Walker and Roberts, 2018[27]). The scientific community at large must demand greater accountability from editors to prevent such inappropriate retroactive decisions from eroding trust in peer-reviewed literature (Siddique et al., 2024[22]). This episode also highlights how egregiously the COPE guidelines can be misapplied and calls into question whether publishing guidelines are effective in protecting scientific integrity, or merely shield editors and publishers from accountability and responsibility. Amidst a waning need for corporate publishers' involvement in scientific communication and growing concerns about their ethical effectiveness, scientists should consider alternative models for refereed publication (e.g., *EXCLI Journal *[EXCLI, 2026[11]]). With the strongest conviction, we assert that retracting a paper without scientific flaws isn't protection-it is censorship. We therefore call for the immediate reversal of this flawed and unjustified retraction.

## Disclosure

This work was originally submitted as a Commentary to *Regulatory Toxicology and Pharmacology* (RTP) as a courtesy to retracting editor Dr. Martin van den Berg, the journal, and the publisher (Elsevier). The piece was withdrawn from RTP because co-Editor-in-Chief Dr. van den Berg would only accept a truncated version that neither transparently listed all authors nor permitted a full exposition of his retraction decision. This work was then invited as a Guest Editorial in *Archives of Toxicology* (AoT), receiving full acceptance in early March 2026. Following acceptance, publication in AoT was repeatedly and unjustifiably delayed due to inappropriate interference by the publisher of that journal (Springer Nature) over the objections of the Editor-in-Chief and Deputy Editor-in-Chief.

The views expressed in this Editorial are solely those of the authors, and they alone are responsible for the decision to seek publication of this work. 56 authors have nothing to disclose. 

AF and CAB report employment during the past 5 years by commercial, private, or public entities that have or could be perceived to have an interest in glyphosate products.

AF, BB, CAB, JD, and TWS report consulting relationships or other professional compensation during the past 5 years related to glyphosate products.

AF, CJB, GB, LDB, OP, and SCB report participation during the past 5 years in regulatory or legal proceedings on behalf of commercial, private or public entities or individuals that have or could be perceived to have an interest in glyphosate products.

### Artificial Intelligence (AI) - assisted technology

Artificial intelligence (AI) was used to augment searching of standard scientific databases for articles relevant to the subject retraction, to assemble the authors' summaries of various issues into an initial rough draft, and to check grammar and spelling.

## Notes

*This Guest Editorial underwent the customary internal review at two journals and an extra-ordinary external review by two referees.*
