# Retraction of the landmark glyphosate safety publication by Williams, Kroes and Munro (2000) should be reversed

**DOI:** 10.17179/excli2026-9686

**Published:** 2026-07-27

**Authors:** Christopher J. Borgert, Mohammad Abdollahi, Jay M. Ansell, Michael Aschner, Christopher A. Bates, Sir Colin Berry, Brad Bolon, Peter J. Boogaard, Robert A. Budinksy, Lyle D. Burgoon, Gary Burin, James S. Bus, Harvey Clewell, Samuel M. Cohen, Thomas Colnot, Paul S. Darby, Wolfgang Dekant, John DeSesso, Daniel R. Dietrich, Mirjana Djukic, Anca Oana Docea, Jose L. Domingo, David L. Eaton, Jeffery A. Engelhardt, Anne Fairbrother, Allan Felsot, Penelope A. Fenner-Crisp, Robinan Gentry, Jay I. Goodman, Gio B. Gori, Helmut Greim, J.S. Heslop-Harrison, Jan G. Hengstler, Hartmut Jaeschke, Norbert E. Kaminski, Sam Kacew, James Kim, Anthony L. Kiorpes, Curtis Klaassen, James E. Klaunig, Anne Loccisano, James S. MacDonald, Angela Mally, Hans W. J. Marquardt, M. Sue Marty, John C. Matthews, Roger O. McClellan, Robert G. Meeks, Glenn C. Millner, Michael W. Pariza, Dennis Paustenbach, Olavi Pelkonen, David Sarvadi, Kai Savolainen, Ted W. Simon, Carr J. Smith, Keith R. Solomon, Stanley M. Tarka, Jr., Aristidis M. Tsatsakis, Joyce Tsuji, Nico P. E. Vermeulen, Kendall B. Wallace, Michael Wernke, Jonathan P. Williams, Raphael J. Witorsch, Hiroshi Yamazaki

**Affiliations:** 1 Applied Pharmacology & Toxicology, Inc., Gainesville, FL, USA; 2 Mohammad Abdollahi, Department of Toxicology and Pharmacology, Tehran University of Medical Sciences, Tehran, Iran; 3 Jay M. Ansell, Principal, Bellevue Toxicology LLC, Alexandria VA, USA; 4 Michael Aschner, Department of Molecular Pharmacology, Albert Einstein College of Medicine, Bronx, NY, USA; 5 Christopher A. Bates, H.B. Fuller, Saint Paul, MN, USA; 6 Sir Colin Berry, Prof. (emeritus) of Pathology, Queen Mary, London, UK; 7 Brad Bolon, Sr. Advisor to the Editor-in-Chief of the journal Toxicologic Pathology, President and Pathologist, GEMpath, Inc., Longmont, CO, USA; 8 Peter J. Boogaard, Editor-in-Chief of Critical Reviews in Toxicology, Professor of Environmental Health & Human Biomonitoring, Division of Toxicology - AFSG, Wageningen University and Research, Wageningen, The Netherlands; 9 Robert A. Budinsky, (retired) The Dow Chemical Company, Midland, MI, USA; 10 Lyle D. Burgoon, Raptor Pharm & Tox, Ltd. Apex, NC, USA; 11 Gary Burin, (retired) Technology Sciences Group, Inc., Vienna, VA, USA; 12 James S. Bus, Exponent, Inc., Wendell, NC, USA; 13 Harvey Clewell, Science to Support Regulatory Activities, Ramboll Americas Engineering Solutions, Inc., Research Triangle Park, NC, USA; 14 Samuel M. Cohen, Department of Pathology, Microbiology and Immunology, College of Medicine, University of Nebraska Medical Center, Omaha, NE, USA; 15 Thomas Colnot, EUROTOX, CiS Toxicology, Osorno, Chile; 16 Paul S. Darby, MD PhD Services, PLLC, Tacoma, WA, USA; 17 Wolfgang Dekant, Prof. (emeritus) of Toxicology, Department of Pharmacology and Toxicology, University of Würzburg, Germany; 18 John DeSesso, Exponent, Inc., Alexandria, VA, USA; 19 Daniel R. Dietrich, Prof. (emeritus) of Toxicology, Department of Human and Environmental Toxicology, University of Konstanz, Germany, Past-President Academy of Toxicological Sciences, Editor-in-Chief of Chemico-Biological Interactions, Computational Toxicology, and the Journal of Toxicology and Regulatory Policy, Konstanz, Germany; 20 Mirjana Djukic, University of Belgrade-Faculty of Pharmacy, Department for Toxicology, Belgrade, Serbia; 21 Anca Oana Docea, Department of Toxicology, University of Medicine and Pharmacy of Craiova, Craiova, Romania; 22 Jose L. Domingo, Distinguished Prof. (emeritus) of Toxicology, Universitat Rovira i Virgili, School of Medicine, Reus, Spain; 23 David L. Eaton, President, Academy of Toxicological Sciences, Prof. (emeritus) School of Public Health, University of Washington, Seattle, WA, Adjunct Professor of Pharmacology & Toxicology, R. Ken Coit College of Pharmacy, University of Arizona, Tucson, AZ, USA; 24 Jeffery A. Engelhardt, (retired) Vice President of Pathology and Nonclinical Development, Ionis, Carlsbad, CA, USA; 25 Anne Fairbrother, (retired), Exponent, Inc., Philomath, OR, USA; 26 Allan Felsot, Department of Entomology & Environmental Toxicology, Washington State University, Richland, WA, USA; 27 Penelope A. Fenner-Crisp, (retired) Environmental Protection Agency, Charlottesville, VA, USA; 28 Robinan Gentry, Science to Support Regulatory Activities, Ramboll Americas Engineering Solutions, Inc., Monroe, LA, USA; 29 Jay I. Goodman, Prof. (emeritus), Department of Pharmacology and Toxicology, Institute for Integrative Toxicology, Michigan State University, East Lansing, MI, USA; 30 Gio B. Gori, Editor-in-Chief (emeritus) of Regulatory Toxicology and Pharmacology(2002 - 2009), The Health Policy Center, Bethesda, MD, USA; 31 Helmut Greim, Prof. (emeritus) of Toxicology, Technical University of Munich, Germany; 32 J.S. Heslop-Harrison, Genetics and Genome Biology, Institute for Environmental Futures, University of Leicester, UK; 33 Jan G. Hengstler, Department of Toxicology, Leibniz Research Centre for Working Environment and Human Factors, Dortmund, Germany; 34 Hartmut Jaeschke, Editor-in-Chief of Livers, Associate Editor of Toxicological Sciences, Distinguished Prof. Department of Pharmacology, Toxicology & Therapeutics, University of Kansas, Kansas City, KS, USA; 35 Norbert E. Kaminski, Department of Pharmacology & Toxicology, Michigan State University East Lansing, MI, USA; 36 Sam Kacew, Editor-in-Chief of Journal of Toxicology and Environmental Health Parts A & B, McLaughlin Centre for Population Health Risk Assessment, Faculty of Medicine, University of Ottawa, ON, Canada; 37 James Kim, Sr. Vice President, Science & Regulatory Affairs, American Cleaning Institute®, Washington, DC, USA; 38 Anthony L. Kiorpes, Principal, River Bluff Associates, LLC, Bloomington, MN, USA; 39 Curtis Klaassen, (retired) Department of Pharmacology, Toxicology and Therapeutics, College of Medicine, University of Kansas, Kansas City, KS, USA; 40 James E. Klaunig, Prof. (emeritus) of Environmental Health and Toxicology, Indiana University, Bloomington, IN, USA; 41 Anne Loccisano, Exponent, Inc., Alexandria, VA, USA; 42 James S. MacDonald, Founding Partner, Synergy Partners R&D Solutions, Chester, NJ, USA; 43 Angela Mally, Department of Toxicology, University of Würzburg, Germany; 44 Hans W. J. Marquardt, Prof. (emeritus) of Toxicology, Department of Experimental and Clinical Pharmacology and Toxicology, University of Hamburg Medical School, Germany; 45 M. Sue Marty, The Dow Chemical Company, Midland, MI, USA; 46 John C. Matthews, Prof (emeritus) University of Mississippi School of Pharmacy, University, MS, USA; 47 Roger O. McClellan, Editor-in-Chief (emeritus) of Critical Reviews in Toxicology (1987 - 2025); Independent Advisor, Toxicology and Human Health Risk Analysis, Albuquerque, NM, USA; 48 Robert G. Meeks, Shin-Etsu Silicones of North America, Sarasota, Florida, USA; 49 Glenn C. Millner, Founder and Principal Toxicologist, Center for Toxicology and Environmental Health (CTEH), LLC, Little Rock, AR, USA; 50 Michael W. Pariza, Prof. (emeritus), Food Science, Director (emeritus), Food Research Institute University of Wisconsin-Madison, WI, USA; 51 Dennis Paustenbach, Toxicology and Risk Exposure, TRC, Jackson, WY, USA; 52 Olavi Pelkonen, Prof. (emeritus) of Pharmacology, Department of Pharmacology and Toxicology, University of Oulu, Finland; 53 David Sarvadi, MS, JD, Bonita Springs, FL, USA; 54 Kai Savolainen, Editor (emeritus) of Human and Experimental Toxicology (2002-2024), Finnish Institute of Occupational Health, Helsinki, Finland; 55 Ted W. Simon, Ted Simon LLC, College of Public Health, University of Georgia, Winston, GA, USA; 56 Carr J. Smith, Society for Brain Mapping and Therapeutics, Mobile Alabama, USA; 57 Keith R. Solomon, Prof. (emeritus), School of Environmental Sciences, University of Guelph, Guelph, ON, Canada; 58 Stanley M. Tarka, Jr., Adjunct Associate Professor, Neuroscience and Experimental Therapeutics, Pennsylvania State University College of Medicine, Hershey, PA, USA; 59 Aristidis M. Tsatsakis, Center of Toxicology and Science Applications, Medical School, University of Crete, Heraklion, Greece; Universidad Ecotec, Health Sciences Dept., Km. 13.5 Samborondón, EC092302, Ecuador; Sechenov IM First State Medical University, Science Technology Park, Moscow 119991, Russia; 60 Joyce Tsuji, Exponent, Inc., Bellevue, WA, USA; 61 Nico P. E. Vermeulen, Prof. (emeritus) of Molecular Toxicology, Department of Chemistry & Pharmaceutical Sciences, VU University Amsterdam, The Netherlands; 62 Kendall B. Wallace, Prof. (emeritus) of Biochemistry & Molecular Biology, University of Minnesota Medical School, Duluth, MN, USA; 63 Michael Wernke, Health Sciences Sr. Toxicologist and Pharmacologist, Sea Limited, Columbus, OH, USA; 64 Jonathan P. Williams, Assoc. Prof., Department of Statistics, North Carolina State University, Raleigh, NC, USA; 65 Raphael J. Witorsch, Prof. (emeritus) of Physiology and Biophysics, School of Medicine, Virginia Commonwealth University, Richmond, Virginia, USA; 66 Hiroshi Yamazaki, Showa Pharmaceutical University, Machida, Tokyo, Japan

## Corrigendum⁯⁯⁯

Corrigendum to Scientific Forum article: https://doi.org/10.17179/excli2026-9644

[EXCLI Journal 2026;25:1107-1116]; PMID: 42517087 ; PMCID: PMC13402740

Dear Editors,

an article we cited in-text (p. 1111, 1112, 1113) and in our reference list (p. 1115) should be removed because the citation information is incorrect and the article is superfluous to the other publications from the lay press that we also cited. The reference that should be deleted from our article is: 

Mozart M (2025). Glyphosate safety article retracted eight years after Monsanto ghostwriting revealed in court. Retraction Watch. https://retractionwatch.com/2025/12/04/glyphosate-safety-article-retracted-elsevier-monsanto-ghostwriting/

Dr. Christopher J. Borgert *(on behalf of all authors)*

Applied Pharmacology & Toxicology, Inc., Gainesville, FL, USA. E-mail: cjborgert@apt-pharmatox.com

## Notes

*This Guest Editorial underwent the customary internal review at two journals and an extra-ordinary external review by two referees.*

